# Association Between Redlining and Spatial Access to Pharmacies

**DOI:** 10.1001/jamanetworkopen.2023.27315

**Published:** 2023-08-04

**Authors:** Giovanni Appolon, Shangbin Tang, Nico Gabriel, Jasmine Morales, Lucas A. Berenbrok, Jingchuan Guo, Inmaculada Hernandez

**Affiliations:** 1 The Herbert Wertheim School of Public Health and Human Longevity Science, University of California San Diego, La Jolla; 2 Division of Clinical Pharmacy, University of California, San Diego, Skaggs School of Pharmacy and Pharmaceutical Sciences, La Jolla; 3 Department of Pharmacy and Therapeutics, University of Pittsburgh School of Pharmacy, Pittsburgh, Pennsylvania; 4 Department of Pharmaceutical Outcomes and Policy, University of Florida College of Pharmacy, Gainesville

## Abstract

This cross-sectional study evaluates whether there is an association between historic redlining and living within 1 or 2 miles of a pharmacy.

## Introduction

Historic redlining was a practice implemented in the 1930s by the Homeowners Loan Corporation that identified areas based on the population risk to default on mortgage.^1^ Previous ecological studies have demonstrated that the detrimental effects of redlining on health outcomes persist in the present.^1^ To our knowledge, no study has evaluated the association between redlining and spatial access to health care, including pharmacies. Pharmacy access is particularly relevant for equity in health care access because pharmacies reach individuals who do not have access to other health care settings.^2^ We conducted a nationwide geographic information systems analysis to estimate the association between residence in a historically redlined neighborhood and spatial access to community pharmacies.

## Methods

In this cross-sectional study, we obtained from Research Triangle Institute (RTI) International a 30% random sample of the 2020 US Synthetic Population (N = 90 769 595). The RTI Synthetic Population comprises statistically accurate records for every household and individual and can be interpreted as census data without identifiers.^3^ We obtained from the University of Michigan Institute for Social Research historic redlining data for 142 cities across the United States,^4^ which we linked to the synthetic population at the census tract level. Our final sample was constrained to synthetic individuals living in census tracts within the 142 cities with available redlining scores (N = 13 009 569). Our study adheres to the STROBE reporting guidelines for cross-sectional studies and, per the Common Rule, is exempt from institutional review board approval and the requirement for informed consent, as no human data were used.

For each synthetic individual, the primary exposure was defined as residence in a redlined census tract. To define the primary outcome variable (residence within 1-mile driving distance of a pharmacy), we obtained addresses for community pharmacies operating on July 1, 2020, from the National Council for Prescription Drug Programs and estimated service areas based on driving distance using OpenStreetMap’s road network data set.^5^ In secondary analyses, we defined pharmacy access as residence within 2 miles’ driving distance.

We performed a multivariate logistic regression to evaluate the association between residence in a redlined area and spatial access to a pharmacy, controlling for age, sex, race and ethnicity, household income, and Area Deprivation Index (ADI). ADI is a measure of socioeconomic disadvantage and was operationalized as an indicator variable denoting residence in a block group within the fourth quartile of ADI (most deprived neighborhoods).^6^ We conducted interaction analyses to test whether the association between residence in a redlined neighborhood differed by ADI. We conducted 2-sided *t* tests with a significance level of .05.

## Results

In the sample of 13 009 569 synthetic individuals, 48.0% were women; 22.6% were Hispanic, 19.4% were non-Hispanic Black, and 45.3% were non-Hispanic White; and 86.2% were younger than 65 years. Overall, 85.4% of the sample lived within 1 mile of a community pharmacy and 98.7% within 2 miles (Table). Residence in a redlined census tract was associated with 9% decreased odds of pharmacy access within 1 mile (Figure, A). Residence in a deprived neighborhood (top quartile of ADI) was associated with 59% decreased odds of pharmacy access within 1 mile (probability of pharmacy access within 1 mile, 76% in ADI quartile 4 vs 88.6% in ADI quartiles 1-3).

**Table.** Sample Characteristics

Characteristic	Individuals, No. in millions (%)		
	Total (N = 13 009 569)	Redlined neighborhood	
		No (N = 9 150 463)	Yes (N = 3 859 106)
Pharmacy access^a^			
Distance ≤1 mile	11.1 (85.4)	7.9 (85.8)	3.3 (84.5)
Distance ≤2 miles	12.8 (98.7)	9.0 (98.8)	3.8 (98.2)
Race and ethnicity			
Hispanic	2.9 (22.6)	1.9 (20.9)	1.0 (26.7)
Non-Hispanic Asian or Hawaiian	0.9 (6.8)	0.6 (7.0)	0.2 (6.4)
Non-Hispanic Black	2.5 (19.4)	1.6 (17.4)	0.9 (24.1)
Non-Hispanic Indigenous	0.2 (1.5)	0.1 (1.4)	0.1 (1.7)
Non-Hispanic White	5.9 (45.3)	4.5 (48.9)	1.4 (36.6)
Other^b^	0.6 (4.5)	0.4 (4.5)	0.2 (4.6)
Income, $			
<25 000	3.2 (24.7)	2.0 (22.3)	1.2 (30.3)
25 000-100 000	6.3 (48.4)	4.5 (48.7)	1.8 (47.7)
>100 000	3.5 (26.9)	2.7 (29.0)	0.9 (47.7)
Gender			
Men	6.2 (48.0)	4.4 (48.5)	1.8 (47.6)
Women	6.8 (52.0)	4.7 (51.8)	2.0 (52.4)
Area Deprivation Index^c^			
Least deprived neighborhoods, Area Deprivation Index quartiles 1-3	9.7 (75.1)	6.9 (76.3)	2.8 (72.2)
Most deprived neighborhoods, Area Deprivation Index quartile 4	3.2 (24.9)	2.1 (23.7)	1.1 (27.8)
Age, y			
<65	11.2 (86.2)	7.8 (85.7)	3.4 (87.5)
≥65	1.8 (13.8)	1.3 (14.3)	0.5 (12.5)

^a^ Access was defined as driving distance of 1 mile or less and 2 miles or less.

^b^ Other includes “some other race” and 2 or more races.

^c^ Area Deprivation Index was categorized in quartiles, the following ADI scores were grouped into each quartile: quartile 1, 1 to 10; quartile 2, 11 to 29; quartile 3, 30 to 69; quartile 4, 70 to 100.

**Figure.**
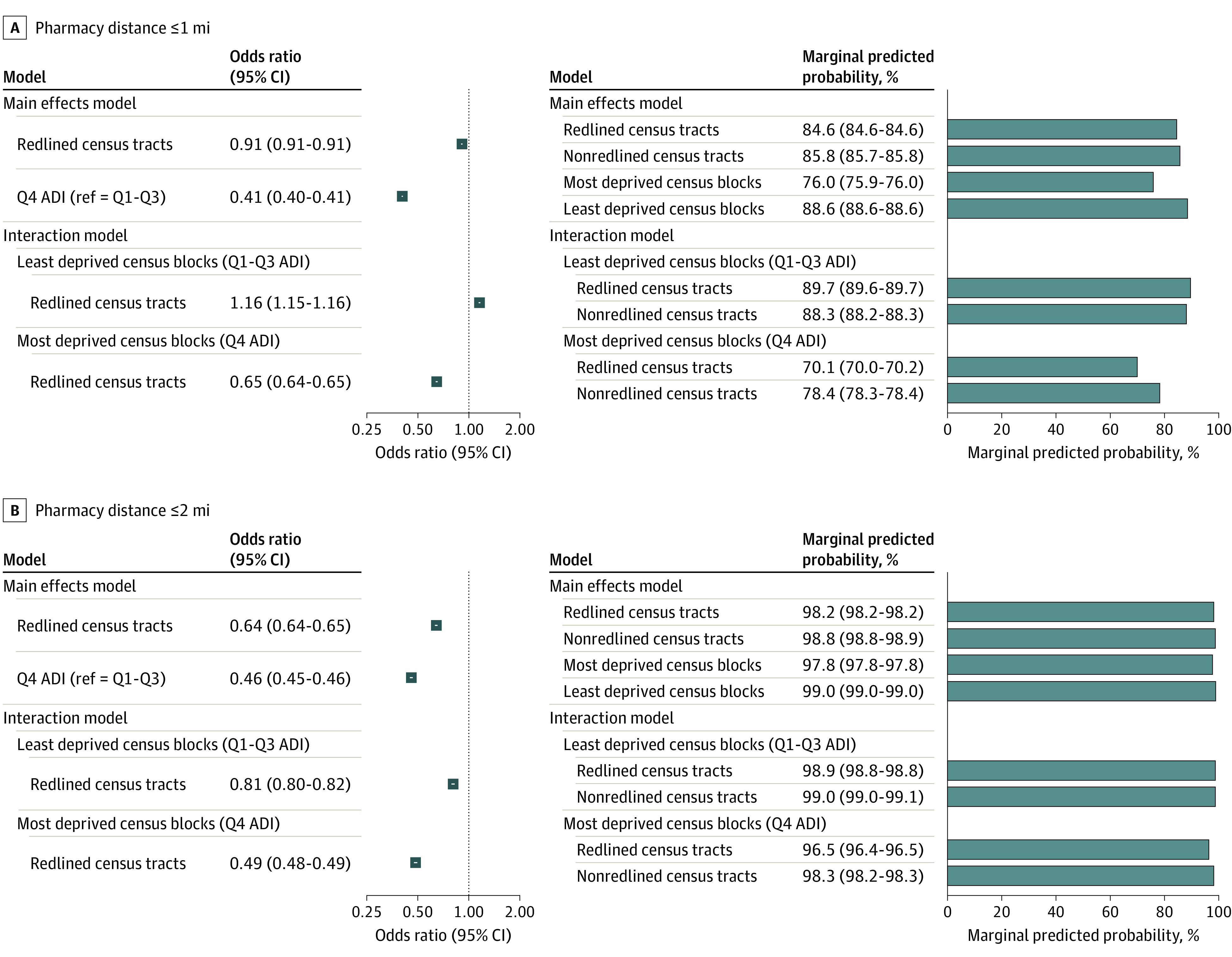
Adjusted Odds Ratios and Marginal Predicted Probability of Optimal Spatial Access The figure shows the odds ratio and marginal predicted probability of optimal pharmacy access, which was estimated by a regression model that regressed optimal spatial access to pharmacies against redlined neighborhoods, controlling for age, race and ethnicity, income, gender, and Area Deprivation Index (ADI). Figure also shows interaction between ADI and redlined neighborhood. Q indicates quartile.

The association of redlining on pharmacy access differed with ADI (*P* for interaction < .001). The consequences of redlining were particularly pronounced in most deprived neighborhoods, where redlining was associated with 35% and 51% decreased odds of living with 1 and 2 miles of a pharmacy, respectively.

## Discussion

To our knowledge, our study is the first nationwide evaluation of the association between historical redlining and spatial access to health care. Our findings are limited by the sole estimation of pharmacy access based on driving distance, which does not account for diversity in modes of transportation. Our nationwide assessment of the association between historical redlining and spatial access to pharmacies provides additional evidence of the detrimental effects redlining continues to have on population health, particularly in socioeconomically deprived neighborhoods. Our findings support the consideration of policies that prevent the closure and incentivize the opening of health care facilities, like community pharmacies, in historically redlined and deprived neighborhoods.

## Notes

Supplement.
Data Sharing Statement

## References

[1] Redlining and neighborhood health. NCRC. Accessed February 26, 2023. https://ncrc.org/holc-health/

[2] Guadamuz JS, Wilder JR, Mouslim MC, Zenk SN, Alexander GC, Qato DM. Fewer pharmacies in Black and Hispanic/Latino neighborhoods compared with White or diverse neighborhoods, 2007-15. Health Aff (Millwood). 2021;40(5):802-811. doi:10.1377/hlthaff.2020.01699 33939507

[3] RTI International. RTI SynthPop. Accessed July 3, 2023. https://www.rti.org/synthpop-synthetic-population-data-analysis

[4] Meier HCS, Mitchell BC. Historic redlining scores for 2010 and 2020 US Census Tracts. Inter-university Consortium for Political and Social Research. Published online October 15, 2021. Accessed February 1, 2023. doi:10.3886/E141121V2

[5] Planet OSM. Accessed January 28, 2023. https://planet.openstreetmap.org/

[6] Kind AJH, Buckingham WR. Making neighborhood-disadvantage metrics accessible—the Neighborhood Atlas. N Engl J Med. 2018;378(26):2456-2458. doi:10.1056/NEJMp1802313 29949490 PMC6051533

