# Supplementary material for: Association Between Redlining and Spatial Access to Pharmacies
Source: JAMA Netw Open. 2023 Aug 4;6(8):e2327315. doi: 10.1001/jamanetworkopen.2023.27315 (PMC10403774; doi:10.1001/jamanetworkopen.2023.27315)
Supplement: Supplement. — Data Sharing Statement [file jamanetwopen-e2327315-s001.pdf]

Data Sharing Statement

Appolon. Association Between Redlining and Spatial Access to Pharmacies. *JAMA Netw Open*. Published August 04, 2023. doi:10.1001/jamanetworkopen.2023.27315

Data
Data available: No
